# Literary Gossip and Record

**Published:** 1861-04

**Authors:** 


### Art. XVII.—LITERARY GOSSIP AND RECORD.

A current bibliography is a desideratum in English medical literature. Its utility would be great. To the reader, the medical scholar, and the librarian it would be a most welcome aid, and its after uses would be as valuable as its present. He alone who has had to hunt up some details in past medical history, general or special, can appreciate the actual value of a trustworthy bibliography. What days, and weeks, and months of profitless labour would have been saved to many a student if he could always have commanded one! And what a gain to medical science! A contemporary bibliography would at once have made apparent every source of information open to the student, and there is no branch of medicine which would not have gained in precision and completeness of detail, in all that relates to the history of its development and progress. Moreover, a current bibliography would prove the best and most certain index to the intellectual workings of a period.

Not the less important would be its present uses. Without one, the knowledge even of those best situated for ascertaining the number, nature, and character of the books published at any given time is imperfect, while those who are not so situated are often left most egregiously in the lurch. A current bibliography, therefore, would be the surest guide to the librarians of our great medical libraries, and would at the same time fill up a very serious hiatus in those libraries. It is, perhaps, not to be desired (although we are somewhat doubtful upon this point) that these should have placed upon their shelves every English medical work published, good, bad, or indifferent. There must doubtless be some restriction in expenditure on the one hand, and on the other, the National Library affords a refuge for all works, irrespective of character. But in the medical libraries it would certainly be no small gain if there could always be found a bibliographical record, so that at any moment the titles and authorship of books not upon the shelves could be ascertained, and thus reference facilitated to the catalogues of the National Library, and any work readily traced out, which, not having been purchased at the time of publication, it might subsequently become an advantage to obtain.

To the literary medical man, particularly when at a distance from the great libraries, the utility of a current bibliography is obvious, enabling him, as it would, to maintain an accurate knowledge of the works issuing from the medical press.

It may be presumed that the *trade* value of a current bibliography would be of less importance than its literary, or we should have had one long ago. Hence, all the more credit is due to a publisher, who, seeing the advantages which would arise to the profession from a bibliography, seeks to supply the want. Now, thanks to the enterprise of Mr. J. W. Davies (our own publisher), we hope soon to be enabled to announce to the profession the publication of a trustworthy current bibliography. He has had the project some time in contemplation, and he hopes soon, we believe, to mature it.

The difficulties are by no means small in carrying out such a scheme, as we know from bitter experience. For we propose, in this and future numbers of our journal, to print a list of the medical books and pamphlets published during the preceding quarter. We have experienced no little difficulty, however, in obtaining either a complete list or the complete titles of works published. The difficulty has been especially great in respect of pamphlets. These *enfans perdus* of authors would seem chiefly to fall from the press still-born. Whether the authors or the publishers are most in fault for this sad ending we shall not presume to judge. It is certain, however, that pamphlets form a most interesting item in the literary history of a period, and that they often contain much valuable matter not given to the world in any other form ; hence it is important that, if possible, a permanent record of their publication should be preserved. We appeal, therefore, to our readers to assist us in this matter, and to help us in rectifying such omissions as may occur from time to time in our bibliographical list.

